# Screening for Attention Deficit Hyperactivity Disorder in Young Autistic Adults: The Diagnostic Accuracy of Three Commonly Used Questionnaires

**DOI:** 10.1007/s10803-023-06146-9

**Published:** 2023-10-28

**Authors:** Melanie Palmer, Zhaonan Fang, Matthew J Hollocks, Tony Charman, Andrew Pickles, Gillian Baird, Emily Simonoff

**Affiliations:** 1 Department of Child and Adolescent Psychiatry, Institute of Psychiatry, Psychology & Neuroscience, King’s College London, London, UK; 2 Service for Complex Autism & Associated Neurodevelopmental Disorders, South London and Maudsley NHS Foundation Trust, London, UK; 3 Department of Biostatistics and Health Informatics, Institute of Psychiatry, Psychology & Neuroscience, King’s College London, London, UK; 4 Newcomen Neurodevelopmental Centre, Evelina Children’s Hospital, Guy’s and St Thomas NHS Foundation Trust, London, UK

**Keywords:** Autism, young adults, ADHD, screening, accuracy

## Abstract

**Objective:**

Attention Deficit Hyperactivity Disorder (ADHD) is a common co-occurring condition in autistic individuals. ADHD is sometimes first recognised in young adulthood because ADHD symptoms may be misattributed to autism due to superficial overlap in presentation and diagnostic overshadowing. It should be investigated whether ADHD questionnaires are accurate in screening symptoms in young adults with autism. The current study examined this.

**Methods:**

Participants were autistic young adults (*N* = 119) who took part in the Special Needs and Autism Project (SNAP), a population-based cohort. ADHD research diagnoses were obtained through the parent-informed Young Adult Psychiatric Assessment. Parents and young adults (self-report sample *N* = 71) completed ADHD questionnaires (Aberrant Behavior Checklist hyperactivity/non-compliance subscale, Conners Adult ADHD Rating Scales ADHD Index, and Strengths and Difficulties Questionnaire ADHD subscale). Receiver operating characteristic analyses were conducted to explore if the questionnaires discriminated ADHD cases from non-cases. To assess whether results varied by intellectual functioning, subgroup analyses were completed for those with an IQ ≥ 70 vs. <70.

**Results:**

Weighted ADHD rates were high. Overall although the measures were performing at or close to adequate levels (area under the curve was 0.66 to 0.79 for parent-report and 0.70 to 0.65 for self-report), no single measure met adequate thresholds for sensitivity and specificity simultaneously. Tool performance was not different for those with an IQ ≥ 70 vs. <70.

**Conclusion:**

No single measure reported adequate performance for distinguishing ADHD from non-ADHD cases in this sample of young autistic adults. Use of current thresholds may lead to under-diagnosis.

## Introduction

Attention Deficit Hyperactivity Disorder (ADHD) is a neurodevelopmental condition characterised by impairing problems with inattention and/or hyperactivity/impulsivity that are beyond what is expected for an individual’s developmental level (American Psychiatric Association, 2013). ADHD is usually recognised in early childhood with symptoms remaining into adulthood (particularly inattention and impulsivity) with some reduction in level (Willcutt et al., 2012; Wootton et al., 2022). Although typically conceived as a childhood-recognised condition, it is now agreed that ADHD can first be recognised in adolescence or young adulthood (Sonuga-Barke et al., 2022).

There are several possible reasons why many individuals with ADHD are not identified until later in life. One possibility is that there may be truly adult-onset cases, although this view is still controversial (Sonuga-Barke et al., 2022). Another possibility is that onset was during childhood, but ADHD symptoms are not recognised until later due to a range of other factors. In support of this there is some evidence that late-recognised ADHD appears to differ slightly from early-recognised ADHD in that symptoms seem less severe, and individuals may have higher intellectual ability (e.g., Asherson & Agnew-Blais, 2019). Given the acknowledgement of late-recognised ADHD, it is important that instruments used for measuring ADHD, mainly developed for use with children, are accurate in identifying symptoms in these age groups. In addition, young adults may be capable of reliably self-reporting on their own symptoms, adding another source of information for clinical consideration.

Autism Spectrum Disorder (hereafter referred to as autism as it is the term preferred by the autistic community, Kenny et al., 2016) commonly co-occurs with ADHD. Autism is characterised by atypicality in social interaction and communication and repetitive, restricted, stereotyped patterns of behaviours and interests (American Psychiatric Association, 2013). ADHD occurs at rates of approximately ~30% in autistic young adults (Lever & Geurts, 2016; Rong et al., 2021). There is evidence of high stability of ADHD symptoms in autistic young people (Carter Leno et al., 2022).

Some autistic individuals with ADHD also do not have their co-occurring condition recognised until late adolescence or young adulthood. One reason is due to diagnostic overshadowing, which may occur when ADHD symptoms are misattributed to other developmental delays (Mason & Scior, 2004), such as autism, because of superficial overlap in symptoms. For example, it can be difficult to differentiate autistic mannerisms from fidgeting, a symptom of ADHD; or lack of understanding of social rules characteristic of autism from displays of impulsivity, such as interrupting conversations. Between childhood and adolescence, Hollocks et al. (2022) found that 16% of autistic young people received an ADHD diagnosis, which may in part be due to diagnostic overshadowing. Another possibility for late recognition may be the increased accuracy in diagnosing individuals with the inattentive type, because they are more capable of describing their own internal states as their age increases. It is therefore important that ADHD screening instruments are accurately picking up ADHD in autistic young adults. Furthermore, as many autistic individuals also have a co-occurring Intellectual Disability (ID) (e.g., Charman et al., 2011; Christensen & Zubler, 2020), understanding how well measures work for autistic individuals with and without ID is important. The overlap in ADHD symptoms and behaviours associated with an ID should also be considered here.

There is some research that has examined how ADHD measures perform as screening instruments in young adults without autism. A systematic review of 35 studies evaluating 14 different ADHD measures with adult populations reported variability in the psychometric properties for different screening instruments (Taylor et al., 2011). They found that self-report and informant-report versions of the Conners Adult ADHD Rating Scales (CAARS) ADHD Index looked adequate with internal consistency of 0.74–0.92, test-retest of 0.80–0.91, and sensitivity of 82% and specificity of 87%. A lower cut point of 4 rather than 6 symptoms was suggested as symptoms weren’t always endorsed to a high level, but impairment existed. However, most studies in the review were of poor quality and insufficiently reported, had small sample sizes, and many did not use a gold standard interview to ascertain ADHD caseness. Taylor et al. (2011) concluded that more research was needed to confirm their findings. Other research exploring the use of a widely used screening instrument for child psychopathology, the Strengths and Difficulties Questionnaire (SDQ), found that the SDQ ADHD subscale had high discriminant validity in distinguishing ADHD cases from non-ADHD cases (AUC = 0.90) in both males and females aged 25 years in a general population cohort, but that a lower cut point (≥ 5) was also needed (Riglin et al., 2021). Therefore, there is emerging evidence that such instruments are effective in picking up ADHD cases in young adults but cut point modification may be required.

Few studies have investigated the accuracy of ADHD measures when it co-occurs with other neurodevelopmental conditions. In one example, Yerys et al. (2017) reported unacceptable model fit for one- (ADHD), two- (hyperactivity/impulsivity vs. inattention), and three-factor (with hyperactivity and impulsivity also separated) solutions for parent and teacher reports of autistic children on the ADHD Rating Scale-IV (ADHD-RS-IV). The least problematic solution was the two-factor solution; however, this included items that were designed to tap into inattention cross-loading onto the hyperactivity/impulsivity factor or vice versa. They suggested that minor changes to item wording was needed when using such instruments with autistic individuals to reduce the influence of autism traits on item endorsement, and that follow-up diagnostic clinical interviews should explore separating inattention from other ADHD symptoms in greater detail. Other research conducted by La Malfa et al. (2008) explored the validity of the Observer: Screener version of the CAARS for assessing ADHD in adults with ID. They found the CAARS had good internal consistency. Scores on the hyperactivity subscale and ADHD index were significantly different across ID severity groups (e.g., mild, moderate, severe, profound), but sample sizes within each group were small limiting conclusions that can be drawn from their findings. The inattentive subscale did not appear to be influenced by ID severity. Taken together, these findings show some promise of ADHD screening instruments across young people with autism and ID, but further evidence of the diagnostic validity of ADHD rating scales is required and to our knowledge, no research has been conducted that has examined the diagnostic validity of ADHD questionnaires in young autistic adults.

The objective of the current study was to examine the discriminant validity of three widely used instruments for distinguishing ADHD from non-ADHD cases in young autistic adults. The measures examined included measures developed for assessing ADHD in the general population (CAARS ADHD Index, SDQ ADHD subscale), in addition to a questionnaire developed for use with individuals with developmental disabilities (the Aberrant Behavior Checklist [ABC] hyperactivity/non-compliance subscale [hereafter referred to as the hyperactivity subscale]). We explored the properties of the different parent- and self-reported versions, and whether the accuracy rates varied by intellectual ability of the young adult.

## Method

### Participants

Participants were autistic young adults and their families who took part in the Special Needs and Autism Project (SNAP). SNAP is a longitudinal study that followed a sample of young people drawn from a population-based cohort of 56,946 in South-East England (see Baird et al., 2006 for further details). The children invited into SNAP were born between July 1990 and December 1991, and either had a clinical diagnosis of autism during the first wave of data collection or were considered to be ‘at risk’ of having autism due to having a statement of Special Educational Needs. A stratified sample of children (*N* = 255) were then assessed for autism using gold standard measures of autism and language, and intellectual and adaptive functioning. Participants in the cohort who received an autism research diagnosis were followed-up when they were young adults at age 23 (Simonoff et al., 2008). Attempts were made to contact all autistic participants. 119 young autistic adults for whom we had ADHD diagnostic data at age 23 and at least one parent-report ADHD measure formed the sample for the current study. Sample characteristics are presented in Table 1 and the participant flow through the study is in the Supplementary Materials.

Written informed consent was obtained from all participating parents and the autistic young adults who had capacity to consent. Where it was suspected that the young adult did not have capacity to consent, a consultee was appointed to determine willingness to participate. Ethical approval for data collection at age 23 was granted by the Camberwell and St. Giles NRES Committee (reference 12/LO/1770, IRAS project number 112286).

### Measures

Demographic information about the young adults and their parents was collected using a questionnaire developed for the study. A measure of neighbourhood deprivation, the Carstairs Index (Carstairs & Morris, 1990), was calculated from full post codes when the autistic individuals were originally assessed at age 12. This index combines overcrowding, male unemployment, population representation in Registrar General social class 4 and 5, and car ownership. Each component is standardised and summed to produce an index which may have positive or negative values. Positive scores indicate greater deprivation. The indices are ordered and grouped into five population quintiles, with quintile 1 representing the least deprived and quintile 5 representing the most deprived in the population.

ADHD diagnoses were obtained through the Young Adult Psychiatric Assessment (YAPA, Angold & Costello, 2000) designed for use with young adults. It is a semi-structured interview administered by a trained researcher/clinician to ascertain detailed descriptions and examples of emotions and behaviours associated with a range of mental health disorders. The modules used in SNAP cover separation anxiety disorder, generalized anxiety disorder, panic disorder, social anxiety, simple phobia, obsessive-compulsive disorder, major depressive disorder, dysthymic disorder, ADHD, oppositional defiant disorder, conduct disorder, Tourette syndrome, chronic tic disorder, trichotillomania, enuresis, and encopresis. The descriptions focus on the intensity, frequency, duration and impairment of symptoms and enable symptoms of different conditions to be disentangled. Use of behavioural descriptions of symptoms decreases the likelihood of diagnostic overshadowing, or incorrectly double coding symptoms, for example, repetitive language associated with autism being incorrectly coded as anxiety and reassurance seeking. In addition, ongoing supervision is used to help interviewers make these distinctions. The YAPA also probes about areas of functioning relevant to this age group, such as living situation and relationships. Standardised algorithms are then applied to generate DSM-V diagnoses and a variety of symptoms and impairment scores. In these analyses we use the parent-reported YAPA for ADHD diagnoses. This was because young adults with lower IQs could not complete the YAPA themselves due to intellectual challenges and we wanted to include the wider group of young autistic adults in the sample to enhance generalisability.

The Aberrant Behavior Checklist (ABC, Aman et al., 1985) was developed specifically to measure a range of problematic behaviours in individuals with developmental disabilities. It is used widely in both clinical and research settings, for example, to assess the effects of psychotropic medication for conditions such as ADHD (e.g., Capone et al., 2016). There is substantial literature examining its validity and reliability with a range of child (e.g., Research Units on Pediatric Psychopharmacology Autism Network, 2005) and adult populations (e.g., Newton & Sturmey, 1988). In the current study, the hyperactivity subscale, which taps into both hyperactive and non-compliant behaviours, was used. The ABC hyperactivity subscale was designed to be completed by someone close to the individual and consists of 16 items (see Supplementary Materials for example items). Items are rated on a 4-point scale ranging from ‘not at all a problem’ to ‘the problem is severe in degree’. Total hyperactivity subscale scores range from 0 to 48.

The Conners Adult ADHD Rating Scales (Conners et al., 1999) is a widely used normed measure of current ADHD symptoms. The Observer: Short version was used in the current study and completed by the young autistic adults and their parents. This version of the CAARS includes items that tap into inattention/memory problems, hyperactivity/restlessness and impulsivity/emotional lability, and self-concept problems. In the current study, responses to the 12 item ADHD Index were used (see Supplementary Materials for example items). Items are rated on a 4-point scale ranging from ‘not at all, never’ to ‘very much, very frequently’. Raw scores are converted to a T score with scores ≥ 60 indicating elevated ADHD problems requiring further assessment.

The Strengths and Difficulties Questionnaire (SDQ, Goodman, 1997) is a questionnaire measure for emotional and behavioural problems in children and young people aged 3–17 years. The SDQ has been used widely as a community screening instrument for child mental health problems (e.g., Goodman et al., 2000), along with screening amongst more vulnerable populations (e.g., Goodman et al., 2004). Although it has not formally been validated in young adults, there is some use with adults (e.g., Riglin et al., 2021). It was used in SNAP as the SDQ had been collected at previous data collection waves when the young adults were children. Five items make up the ADHD subscale which was used in the current study (see Supplementary Materials for example items) and completed by the young autistic adults themselves and their parents. Each item is rated on a 3-point rating scale from ‘not true’ to ‘certainly true’, with a possible range of 0–10.

Intellectual functioning was assessed through the Wechsler Abbreviated Scale of Intelligence (WASI-II, Wechsler, 2011), an abbreviated measure designed to test intellectual ability in individuals aged 6–90 years. The two-subset WASI-II (measuring vocabulary and matrix reasoning) was administered to the first 10 participants, after which participants received the four-subset version (measuring vocabulary, similarities, block design and matrix reasoning) to ensure a comprehensive accurate measure of intellectual functioning. When standard IQ scores showed a floor effect (IQ < 40), a ratio variable was generated by dividing the sum of subscale raw scores by age in months. A regression equation then predicted IQ from the ratio score, the total IQ raw score and age in months for the entire sample, where those with IQ < 40 were set to missing. Fitted residuals from this regression equation then provided IQ estimates for those with IQ < 40. When standard IQ scores were missing, WASI-II scores were imputed using parent-reported Adaptive Behavior Assessment System-II General Adaptive Composite scores (Harrison & Oakland, 2003) as the auxiliary variable (see Supplementary Materials for further information about this procedure). Two IQ subgroups were used in the current study: those with an IQ ≥ 70 and those with an IQ < 70.

### Statistical Analysis

Analyses were completed in Stata version 17.0 (StataCorp., 2021). The participants who were included in the samples for which we had YAPA assessments and parent-/self-report ADHD measures were not mutually exclusive. Attrition at 23 was explored by comparing the parent-/self-report samples that were available for the current analysis to the original autism sample (*N* = 158) for drop out by child sex, parental education, and social deprivation (see Supplementary Materials for participant flow).

The accuracy of each measure was assessed using receiver operating characteristic (ROC) analyses for discriminating cases from non-cases. The resulting area under the curve (AUC) values range from 0 to 1 with an AUC of 1 indicating perfect discrimination and AUC of 0.5 meaning that the scale is not able to discriminate better than chance (Hanley & McNeil, 1982; Mandrekar, 2010). Sensitivity and specificity were calculated for established cut points where applicable (e.g., SDQ ADHD subscale) and for optimally identified cut points using the Youden’s Index (*J*) method (Youden, 1950) commonly used for assessing instrument detection properties which assumes equal weighting for both sensitivity and specificity. Sensitivity values of between 0.7 and 0.8 and specificity of 0.8 are typically regarded as acceptable when screening child psychiatric conditions (Glascoe, 2005). The ROC AUC for the different parent-report and self-report measures were compared. Where sample size allowed (e.g., for parent, but not self-report as the number of young adults in the IQ < 70 [*n* = 10] completed the SDQ ADHD subscale and CAARS ADHD Index vs. IQ ≥ 70 [*n* = 64]), the analyses were also conducted by IQ subgroups. AUC, sensitivity and specificity statistics were bootstrapped to obtain 95% confidence intervals (*CI*) using 1,000 repetitions. All analyses were weighted using frequency weights which consider the original sample stratification and characteristics, along with subsequent attrition in young adulthood. This means that estimates are applicable to the original population from which the sample is drawn. For the ROC analyses, this was done using frequency weights that in theory could generate occasional replicates with out-of-range AUC values (e.g., values of greater than 1) which have not been corrected. Prevalence of ADHD rates were weighted using population weights implemented by the *svy* procedure. All other descriptive analyses were unweighted and therefore represent the characteristics of the sample in the study at young adulthood.

## Results

### Attrition at Young Adulthood

The samples for which we had YAPA assessments and parent-/self-report ADHD measures did not differ from the original autism sample (*N* = 158) by child sex (parent-report sample: *χ*^2^[1, *N* = 158] = 0.00, *p* = .975, self-report sample: *χ*^2^[1, *N* = 158] = 0.18, *p* = .668), but for those who remained in the study, the parents were significantly more educated (N.B. for self-report sample only; parent-report sample: *χ*^2^[1, *N* = 158] = 1.93, *p* = .165, self-report sample: *χ*^2^[1, *N* = 158] = 4.66, *p* = .031), and the families less socially deprived (parent-report sample: *t*[156] = 2.38, *p* = .018, self-report sample: *t*[156] = 2.11, *p* = .036).

### Prevalence of ADHD Cases

In young adulthood, 40 participants meet YAPA ADHD diagnosis for the parent-report sample and 20 amongst those for which we had self-report measures. This equated to weighted population estimates of 22.5% and 23.7% respectively, with an average of 4 symptoms being endorsed (see Table 2). Weighted population estimates for those in the parent-report sample with an IQ < 70 was 41.3% and 15.4% for those with an IQ of ≥ 70. The numbers of young adults who had combined, predominantly inattentive, and predominantly hyperactive ADHD diagnoses respectively for the parent-report sample were: 13 (32.5% of the 40 with ADHD), 12 (30.0% of those with ADHD) and 15 (37.5% of those with ADHD). This equated to weighted population estimates of 9.1%, 6.4%, and 7.1% for the combined, predominantly inattentive, and predominantly hyperactive ADHD diagnoses respectively. Amongst the 71 in the self-report sample 8, 8, and 4 had combined, predominantly inattentive, and predominantly hyperactive ADHD diagnoses respectively (40.0%, 40.0%, and 20.0% of those in the self-report sample with ADHD diagnoses).

### Accuracy of Parent-Report ADHD Measures

Table 3 displays the AUC, sensitivity, specificity, and correctly classified values and their 95% *CI*s for the ADHD measures. Results are broken down into IQ subgroups and presented for optimal and pre-existing cut points where applicable. ROC curves are in the Supplementary Materials, along with the unbootstrapped reports of sensitivity and specificity for all cut points in the data. The overall AUCs for the parent-report measures were: ABC hyperactivity subscale 0.66 (95% *CI* 0.47–0.86), CAARS ADHD Index 0.78 (95% *CI* 0.61–0.94), and SDQ ADHD subscale 0.79 (95% *CI* 0.66–0.92). There were no statistically significant differences in the AUC values between the three parent-report measures, *z*-scores ranged from − 0.13 to 0.31, *p*’s ranged from 0.753 to 0.947. Nor were there any statistically significant differences in instrument performance for those with an IQ < 70 vs. IQ ≥ 70 (ABC hyperactivity subscale: AUC = 0.77 vs. 0.66, *z* = 1.14, *p* = .254; CAARS ADHD Index: AUC = 0.71 vs. 0.76, *z*=-0.10, *p* = .920; SDQ ADHD subscale: AUC = 0.74 vs. 0.75, *z* = 0.03, *p* = .978).

For the ABC hyperactivity subscale and CAARS ADHD Index, sensitivity was high (above 90%), but specificity was inadequate at the *J* optimally identified cut-point for those with an IQ < 70 and IQ ≥ 70 (ABC hyperactivity subscale: cut points of ≥ 4 and ≥ 3 respectively, and CAARS ADHD Index: cut points of ≥ 60 and ≥ 53). Using the pre-existing cut point of T score > 60 for the CAARS ADHD Index improved specificity for the whole sample (from 0.57 to 0.78), but reduced sensitivity (from 0.94 to 0.43). This pattern was consistent across IQ < 70 and IQ ≥ 70 subgroups, but more pronounced in the IQ ≥ 70 subgroup. In contrast, the parent-reported SDQ ADHD subscale demonstrated high specificity (93%), but sensitivity was low (60%) overall. This held across the IQ subgroups and for both the optimal (≥ 7) and pre-existing (≥ 8) cut points. The correct classification rates for the optimally identified cut points were 55% on the ABC hyperactivity subscale, 66% on the CAARS ADHD Index, and 86% on the SDQ ADHD subscale. To achieve 90% specificity required cut points of ≥ 9 on the ABC hyperactivity scale, ≥ 67 T score on the CAARS ADHD Index, and ≥ 7 on the SDQ ADHD subscale (see Supplementary Materials).

### Accuracy of Self-Report ADHD Measures

The AUC, sensitivity, specificity, and correctly classified values and their 95% *CI*s for the self-report ADHD measures are also in Table 3. The ROC graphs are in the Supplementary Materials with the unbootstrapped reports of sensitivity and specificity for all cut points in the data. The AUC for the CAARS ADHD Index and the SDQ ADHD subscale was 0.70 (95% *CI* 0.51–0.90) and 0.65 (95% *CI* 0.44–0.87) respectively. For both the CAARS ADHD Index and the SDQ ADHD subscale, sensitivity was inadequate, although specificity was acceptable for both the optimal cut points (CAARS ADHD Index ≥ 56: 57% and 81% respectively, SDQ ADHD subscale ≥ 9: 28% and 100%) and pre-existing cut points (CAARS ADHD Index > 60: 36% and 90% respectively, SDQ ADHD subscale ≥ 7: 31% and 88%). The correct classification rates at the optimally identified cut point was 74% on the CAARS ADHD Index and 84% the SDQ ADHD subscale. To achieve 90% specificity, a cut point of ≥ 62 on the CAARS ADHD Index ≥ 8 on the SDQ ADHD subscale would be required.

## Discussion

The current study examined the accuracy of parent- and self-reports of ADHD symptoms on three widely used measures for identifying ADHD cases from non-ADHD cases in young autistic adults. The accuracy of the measures was also compared across IQ subgroups to assess whether performance varies by this individual characteristic. Given the acknowledgement of late-recognised ADHD, it is important that instruments used for measuring ADHD are accurate in identifying symptoms in these age groups. Furthermore, due to diagnostic overshadowing, it may be more likely for co-occurring diagnoses to go undetected early in childhood amongst autistic individuals, with a co-occurring condition being identified later.

The AUC statistics indicate that overall, the measures were performing at or close to adequate levels. This is a key finding given that none of these measures were developed for screening of ADHD symptoms with autistic young adults. Parent-report measures appeared to perform similarly across both young autistic adults with IQs above and below 70, suggesting that the measures have similar accuracy for those with and without a co-occurring ID and are appropriate for use across the intellectual ability spectrum. These findings reflect parallel analyses completed by the authors using a sample of autistic children with and without ADHD and ID (in preparation). Although YAPA ADHD diagnoses were made based on parent-reports, self-reported ADHD symptoms from the young autistic adults themselves resulted in similar accuracy statistics to parent-report (N.B. this was a more restricted sample of those who could complete self-report measures). However, although overall the measures were performing adequately, no single measure met adequate thresholds for sensitivity and specificity simultaneously, and there was vast variation in sensitivity and specificity as sensitivity decreased. The current results indicate that there is utility in starting with a broad, short measure, such as the SDQ, which appears to be sufficient for ruling out non-ADHD cases in autistic young adults. In clinical practice, a balance between reducing stress associated with false positive screens, and the costs of false negatives should be considered. The high prevalence of ADHD in autistic individuals should also be taken into account to ensure that cases are not missed and below threshold results on a screening instrument should not be used to exclude consideration of the diagnosis. For research, a more stringent threshold for screening could be taken to ensure that samples include true ADHD cases.

It is important to note that the measures used in the current study were developed for different reasons with a range of populations in mind. The ABC was originally developed to track treatment outcomes, including ADHD symptoms, amongst adults with developmental disabilities (Aman et al., 1985). The CAARS ADHD Index was developed as a tool to assess the presence and severity of ADHD symptoms for use with adults in the general population, whereas the SDQ ADHD subscale was developed as a short questionnaire to be used as a screening instrument for the general child and adolescent population, in which identifying ADHD symptoms is viewed as particularly important. However, all are widely used in the United Kingdom and Europe, and it is important for clinicians to understand their accuracy in autistic populations.

As this is, to our knowledge, the first study exploring the accuracy of ADHD measures in young autistic adults, more research with different samples is required before recommendations about how to use such measures can be made. For example, the modification of cut points may be required for autistic young adults as suggested by research using samples of non-autistic adults (e.g., Riglin et al., 2021; Taylor et al., 2011), with lower cut points needed. However, the balance between a screener’s specificity and sensitivity should be considered (Trevethan, 2017). For example, the optimally identified cut point of 3–4 on the ABC hyperactivity subscale in this study may not be appropriate as this could lead to high false negative rates. This cut point would be equivalent to one symptom being endorsed to the highest degree, or four symptoms being endorsed as a mild level, which may not adequately identify the young adults with impairing ADHD symptoms. This low cut point identified in the current study could be related to the original purpose of the ABC hyperactivity subscale being a treatment monitoring measure, that it is not mapped to DSM ADHD diagnostic criteria, and that it also includes items about non-compliant behaviour. As can be seen in Supplementary Tables 3, which presents the unbootstrapped data for the parent-reported ABC hyperactivity subscale for all cut points observed in the data, the cut point of ≥ 19 would correctly classify the highest proportion of the sample at 77.9%. This cut point is similar to mean ABC hyperactivity subscale scores reported elsewhere in a sample of autistic young people (*M*age = 10.6 years; 14.6 and 23.6 for those with high and low self-injury respectively) (Brinkley et al., 2007). However, other research has reported lower mean scores of 4.8 on the ABC hyperactivity subscale amongst 18–25-year-olds with Fragile X (Wheeler et al., 2014). Alternatively, utilisation of cut points that classify with 90% sensitivity could be adopted to ensure that cases are not missed. Another area of potential modification that should be tested in future research is item adaptation.

### Limitations

The YAPA assessment used to derive ADHD diagnoses for the young adults has not been validated in autistic populations, so it is unclear how autism may influence reports on the YAPA. However, to our knowledge, no other psychiatric research interviews have been validated in this population. Furthermore, as ADHD diagnoses in the current study are based on a research interview, they do not equate to clinical diagnosis which may not account for other contextual factors, such as substance misuse. However, interviewers administering the YAPA are trained to obtain descriptions of emotions and behaviours so that symptoms of different conditions can be disentangled. Youden’s Index (*J*) method was used to identify optimal cut points, which places equal weighting on both sensitivity and specificity. This is just one method for balancing the properties of a measure’s accuracy to detect cases and in screening whole populations, one might place more weight on sensitivity to reduce false negatives. In addition, due to small cell sizes, we were unable to examine the performance of the self-reported ADHD screening instruments by IQ subgroups so we cannot generalise the accuracy reported for the self-report measures to autistic young adults with impairments in intellectual functioning. Further research should explore the performance of such measures for this group, as it is likely adaptation is needed for individuals with IQs < 70 to access such assessments. For example, this could involve using pictorial ratings scales to simplify response options.

## Strengths and Conclusions

The study examined the accuracy of three widely used ADHD screening instruments amongst a well-characterised sample of young autistic adults with and without ADHD and ID. It included reports from parents as well as the young adults themselves and evaluates accuracy across those with a range of intellectual functioning making the findings more generalisable to the autism population. Although the measures were performing adequately overall for both those with IQs of < 70 and IQ ≥ 70, no single measure met satisfactory thresholds for sensitivity and specificity simultaneously. The potential remains to amend measures for better performance in autistic populations; for example, cut point reduction or changes to the wording of individual items may improve accuracy. Clinical use of ADHD screening instruments alone should not be used to determine who receives a diagnostic assessment for young autistic adults and such measures should be supplemented by clinical observation. Clinical judgement should be used to consider adjustments applicable to this population, for example, superficial overlap with autism symptoms should be taken into account and possibly less weight should be placed on these items.

**Table 1:** Sample characteristics

Characteristic		Sample with parent report and YAPA assessment (*N* = 119)	Sample with self report and YAPA assessment (*N* = 71)
**Young adult**			
Mean age in years (*SD*), range		23.10 (0.77), 21.33–25.08	23.00 (0.81), 21.33–25.08
Sex: *n* (%) male		107 (89.9%)	63 (88.7%)
Ethnicity: *n* (%) white		112 (94.1%)	67 (94.4%)
Mean IQ (*SD*), range		84.23, (24.81), 28.37–124^a^	93.17 (19.42), 41.99–124
IQ group: *n* (%) IQ < 70		29 (28.7%)	10 (14.1%)
**Parent/family**			
Parental education: A-levels and above: *n* (%)		73 (61.3%)	48 (67.1%)
Mean Carstairs index (*SD*), range		-0.93 (2.36), -4.31–6.68	-1.11 (2.23), -4.29–6.51
Carstairs quintiles: *n* (%)			
	Quintile 1 (least deprived)	27 (22.7%)	16 (22.5%)
	Quintile 2	27 (22.7%)	18 (25.4%)
	Quintile 3	31 (26.1%)	19 (26.8%)
	Quintile 4	21 (17.7%)	12 (16.9%)
	Quintile 5 (most deprived)	13 (10.9%)	6 (8.5%)

*Note.* Parent education reports are from the original wave of data collection when the children were 12 years. British A-levels are roughly equivalent to American High School Advanced Placement

^a^*N*=101

**Table 2:** Scores on the ADHD measures

Characteristic	Sample with parent report and YAPA assessment	Sample with parent report and YAPA assessment	Sample with parent report and YAPA assessment	Sample with self report and YAPA assessment
	IQ < 70	IQ ≥ 70	(*N* = 119)	(*N* = 71)
	*n* = 29	*n* = 72		
YAPA ADHD diagnosis: *N* (weighted %) present	14 (weighted 41.3%)	20 (weighted 15.4%)	40 (weighted 22.5%)	20 (weighted 23.7%)
YAPA ADHD mean symptoms (*SD*), range	5.59 (3.90), 0–14	3.88 (3.45), 0–15	4.39 (3.60), 0–15	4.21 (3.76), 0–15
Mean ABC hyperactivity subscale scores (*SD*), range	9.57 (9.86), 0-45^a^	4.38 (5.83), 0-34^b^	6.60 (8.49), 0-45^c^	
Mean CAARS ADHD Index T-scores (*SD*), range	56.37 (10.63), 37-82^d^	51.46 (9.72), 35-82^e^	53.39 (10.75), 33–85^f^	51.69 (10.01), 36–79^e^
Mean SDQ ADHD subscale scores (*SD*), range	6.21 (2.24), 2–10	4.58 (2.62), 0–10	5.11 (2.62), 0–10^ g^	4.38 (2.50), 0–10

*Note.* IQ subgroup comparison had smaller sample due to availability of the YAPA, the parent-report measure, and IQ test all required for these analyses

^a^*N*=28; ^b^*N*=71; ^c^*N*=116; ^d^*N*=27; ^e^*N*=70; ^f^*N*=114; ^g^*N*=11

**Table 3:** Bootstrapped area under the curve, sensitivity, specificity, and correctly classified for parent- and self-reported ADHD measures

Measure	Sample	Weighted AUC (95% *CI*)	Cut point	Weighted sensitivity (95% *CI*)	Weighted specificity (95% *CI*)	Weighted correctly classified (95% *CI*)
Parent ABC hyperactivity subscale	Whole sample	0.66 (0.47–0.86)	≥ 3 (optimal)	0.91 (0.79–0.98)	0.42 (0.22–0.68)	0.55 (0.35–0.76)
	IQ < 70	0.77 (0.52–1.01)	≥ 4 (optimal)	0.96 (0.84-1.00)	0.55 (0.26–0.77)	0.73 (0.55–0.87)
	IQ ≥ 70	0.55 (0.26–0.84)	≥ 3 (optimal)	0.86 (0.68-1.00)	0.39 (0.20–0.69)	0.47 (0.27–0.74)
Parent CAARS ADHD index	Whole sample	0.78 (0.61–0.94)	≥ 53 (optimal)	0.94 (0.86-1.00)	0.57 (0.34–0.80)	0.66 (0.45–0.85)
			> 60^a^ (pre-existing)	0.43 (0.25–0.63)	0.78 (0.51–0.97)	0.70 (0.50–0.87)
	IQ < 70	0.71 (0.47–0.95)	≥ 60 (optimal)	0.55 (0.27–0.83)	0.92 (0.79–0.99)	0.78 (0.61–0.92)
			> 60^b^ (pre-existing)	0.51 (0.23–0.78)	0.95 (0.86-1.00)	0.78 (0.58–0.92)
	IQ ≥ 70	0.73 (0.47–0.99)	≥ 53 (optimal)	0.93 (0.80-1.00)	0.60 (0.33–0.85)	0.66 (0.41–0.87)
			> 60^a^ (pre-existing)	0.43 (0.20–0.66)	0.76 (0.48–0.98)	0.70 (0.46–0.90)
Young adult CAARS ADHD index	Whole sample	0.70 (0.51–0.90)	≥ 56 (optimal)	0.57 (0.23–0.81)	0.81 (0.63–0.92)	0.74 (0.57–0.85)
			> 60^a^ (pre-existing)	0.36 (0.03–0.66)	0.90 (0.80–0.97)	0.75 (0.62–0.87)
Parent SDQ ADHD subscale	Whole sample	0.79 (0.66–0.92)	≥ 7 (optimal)	0.60 (0.41–0.80)	0.93 (0.85–0.98)	0.86 (0.76–0.92)
			≥ 8 (pre-existing)	0.45 (0.27–0.65)	0.97 (0.92–0.99)	0.85 (0.76–0.92)
	IQ < 70	0.74 (0.46–1.02)	≥ 7 (optimal)	0.59 (0.32–0.91)	0.92 (0.78–0.98)	0.80 (0.61–0.93)
			≥ 8 (pre-existing)	0.46 (0.21–0.78)	0.96 (0.89-1.00)	0.78 (0.59–0.92)
	IQ ≥ 70	0.73 (0.56–0.91)	≥ 7 (optimal)	0.67 (0.46–0.85)	0.94 (0.85–0.98)	0.89 (0.80–0.95)
			≥ 8 (pre-existing)	0.47 (0.26–0.67)	0.97 (0.92-1.00)	0.89 (0.78–0.94)
Young adult SDQ ADHD subscale	Whole sample	0.65 (0.44–0.87)	≥ 9 (optimal)	0.28 (0.04–0.54)	1.00 (1.00–1.00)	0.84 (0.71–0.92)
			≥ 7 (pre-existing)	0.31 (0.06–0.58)	0.88 (0.73–0.97)	0.75 (0.58–0.87)

*Note.* Since participants did not necessarily have a score of 61, cut point ^a^≥62 or ^b^≥63 was used instead to represent the pre-existing cut point for CAARS ADHD Index. Weighting for AUC in Stata allows for out-of-range values (i.e., values of greater than 1.00) which have not been corrected
